# Supplementary material for: Urban rats as carriers of invasive Salmonella Typhimurium sequence type 313, Kisangani, Democratic Republic of Congo
Source: PLoS Negl Trop Dis. 2022 Sep 6;16(9):e0010740. doi: 10.1371/journal.pntd.0010740 (PMC9481155; doi:10.1371/journal.pntd.0010740)
Supplement: S1 Table — (DOCX) [file pntd.0010740.s001.docx]

**Supplemental Table 1: ENA depository genomes**

| Strain Name | SAMPLE | EXPERIMENT | RUN |
| --- | --- | --- | --- |
| 5232/4 | ERS12335258 | ERX9447049 | ERR9904214 |
| 5248/4 | ERS12335259 | ERX9447050 | ERR9904215 |
| 5318/4 | ERS12335260 | ERX9447051 | ERR9904216 |
| 5353/4 | ERS12335261 | ERX9447052 | ERR9904217 |
| 5376/4 | ERS12335262 | ERX9447053 | ERR9904218 |
| 5390/4 | ERS12335263 | ERX9447054 | ERR9904219 |
| 5403/4 | ERS12335264 | ERX9447055 | ERR9904220 |
| 5464/4 | ERS12335265 | ERX9447056 | ERR9904221 |
| 5548/4 | ERS12335266 | ERX9447057 | ERR9904222 |
| 5566/4 | ERS12335267 | ERX9447058 | ERR9904223 |
| 5568/4 | ERS12335268 | ERX9447059 | ERR9904224 |
| 5578/4 | ERS12335269 | ERX9447060 | ERR9904225 |
| 5590/4 | ERS12335270 | ERX9447061 | ERR9904226 |
| 5598/4 | ERS12335271 | ERX9447062 | ERR9904227 |
| 5601/4 | ERS12335272 | ERX9447063 | ERR9904228 |
| 5699/4 | ERS12335273 | ERX9447064 | ERR9904229 |
| 5809/4 | ERS12335274 | ERX9447065 | ERR9904230 |
| 5810/4 | ERS12335275 | ERX9447066 | ERR9904231 |
| 5835/4 | ERS12335276 | ERX9447067 | ERR9904232 |
| 5899/4 | ERS12335277 | ERX9447068 | ERR9904233 |
| 6014/4 | ERS12335278 | ERX9447069 | ERR9904234 |
| 6213/4 | ERS12335279 | ERX9447070 | ERR9904235 |
| 6284/4 | ERS12335280 | ERX9447071 | ERR9904236 |
| 6332/4 | ERS12335281 | ERX9447072 | ERR9904237 |
| 6436/4 | ERS12335282 | ERX9447073 | ERR9904238 |
| 7279/4 | ERS12335283 | ERX9447074 | ERR9904239 |
| 7292/4 | ERS12335284 | ERX9447075 | ERR9904240 |
| AB 005 FO A1 | ERS12335285 | ERX9447076 | ERR9904241 |
| AB 005 RA A1 | ERS12335286 | ERX9447077 | ERR9904242 |
| AB 006 FO A3 | ERS12335287 | ERX9447078 | ERR9904243 |
| MC 025 FO A1 | ERS12335288 | ERX9447079 | ERR9904244 |
| MC 025 FO A2 | ERS12335289 | ERX9447080 | ERR9904245 |
| MC 025 RA A1 | ERS12335290 | ERX9447081 | ERR9904246 |
| MC 025 RA A3 | ERS12335291 | ERX9447082 | ERR9904247 |
| MC 048 RA A1 | ERS12335292 | ERX9447083 | ERR9904248 |
| MC 053 RA A1 | ERS12335293 | ERX9447084 | ERR9904249 |
| MC 054 FO A1 | ERS12335294 | ERX9447085 | ERR9904250 |
| MC 071 FO A1 | ERS12335295 | ERX9447086 | ERR9904251 |
| MC 071 FO B5 | ERS12335296 | ERX9447087 | ERR9904252 |
| MC 071 RA A2 | ERS12335297 | ERX9447088 | ERR9904253 |
| MC 071 RA B1 | ERS12335298 | ERX9447089 | ERR9904254 |
| MY 100 FO A1 | ERS12335299 | ERX9447090 | ERR9904255 |
| MY 100 RA B2 | ERS12335300 | ERX9447091 | ERR9904256 |
| MY 101 RA A1 | ERS12335301 | ERX9447092 | ERR9904257 |
| MY 102 RA A1 | ERS12335302 | ERX9447093 | ERR9904258 |
| MY 213 RA A1 | ERS12335303 | ERX9447094 | ERR9904259 |
| MY 218 FO A2 | ERS12335304 | ERX9447095 | ERR9904260 |
| MY 300 FO A2 | ERS12335305 | ERX9447096 | ERR9904261 |
| MY 302 RA A1 | ERS12335306 | ERX9447097 | ERR9904262 |
| MY 305 FO A1 | ERS12335307 | ERX9447098 | ERR9904263 |
| MY 365 SE B4 | ERS12335308 | ERX9447099 | ERR9904264 |
| MY 373 FO A3 | ERS12335309 | ERX9447100 | ERR9904265 |
| MY 375 SA A1 | ERS12335310 | ERX9447101 | ERR9904266 |
