# Supplementary material for: Urban rats as carriers of invasive Salmonella Typhimurium sequence type 313, Kisangani, Democratic Republic of Congo
Source: PLoS Negl Trop Dis. 2022 Sep 6;16(9):e0010740. doi: 10.1371/journal.pntd.0010740 (PMC9481155; doi:10.1371/journal.pntd.0010740)
Supplement: S2 Table — (DOCX) [file pntd.0010740.s002.docx]

**Supplemental Table 2.** Morphometric parameters of rats (*Rattus rattus* and *Rattus norvegicus*) related to the *Salmonella* carriage status in Kisangani 2016 – 2018. IQR: interquartile range. There were no significant differences between groups.

|  | *Salmonella* carrier rats  (all serotypes)  (n = 46) | *Salmonella* Typhimurium ST313 carrier rats  (n = 3) | Rats with no *Salmonella* detected (n = 519)* |
| --- | --- | --- | --- |
| Numbers of female rats (%) | 26 (56.5 %) | 2 | 269 (51.8 %) |
| Median length in cm (IQR) | 41.0 (39 - 43) | 42.4 (36.3 – 47.5) | 42.0 (39 - 44) |
| Median length (without tail) in cm (IQR) | 22.2 (13.0 – 25.8) | 22.9 (22.5 – 24.9) | 22.1 (8.3 – 28.4) |
| Median mass in g (IQR) | 280 (105 - 450) | 290 (250 – 450) | 280 (30 - 515) |

* Data from 1 rat were missing
