# Supplementary material for: Urban rats as carriers of invasive Salmonella Typhimurium sequence type 313, Kisangani, Democratic Republic of Congo
Source: PLoS Negl Trop Dis. 2022 Sep 6;16(9):e0010740. doi: 10.1371/journal.pntd.0010740 (PMC9481155; doi:10.1371/journal.pntd.0010740)
Supplement: S3 Table — (DOCX) [file pntd.0010740.s003.docx]

**Supplemental Table 3:** Antibiotic susceptibility data of human and rat *Salmonella* isolates

| **Isolate number** | **Human/**  **rat** | **Date of Sampling** | **Specimen** | **Final ID** | **Ampicillin disk diameter** | **Co-Trimoxazole disk diameter** | **Ceftriaxone disk diameter** | **Ceftazidime disk diamater** | **Chloramphenicol disk diameter** | **Pefloxacin disk diameter** | **Nalidixic acid disk diameter** | **Gentamicin disk diameter** | **Tetracycline disk diameter** | **Meropenem disk diameter** | **Sulphonamide disk diameter** | **Trimethoprim disk diameter** | **Azithromycin e-test MIC** | **Ciprofloxacin e-test MIC** |
| --- | --- | --- | --- | --- | --- | --- | --- | --- | --- | --- | --- | --- | --- | --- | --- | --- | --- | --- |
| 4930/4 | Human | 30/10/2015 | Blood | *Salmonella* Typhimurium | 10 | 10 | 29 | 30 | 12 | 26 | 14 | 24 | 28 | 34 | 10 | 10 | 0,75 | 0,032 |
| 5216/4 | Human | 11/05/2016 | Blood | *Salmonella* II:42:r:- | 25 | 30 | 32 | 29 | 23 | 31 | 26 | 24 | 24 | 36 | ND | ND | 3 | 0,008 |
| 5232/4 | Human | 22/05/2016 | Blood | *Salmonella* Typhimurium | 10 | 10 | 28 | 28 | 10 | 27 | 24 | 10 | 10 | 36 | ND | ND | 2 | 0,012 |
| 5248/4 | Human | 26/05/2016 | Blood | *Salmonella* Typhimurium | 10 | 10 | 32 | 28 | 10 | 28 | 24 | 10 | 10 | 35 | ND | ND | 3 | 0,016 |
| 5307/4 | Human | 01/07/2016 | Blood | *Salmonella* species | ND | ND | ND | ND | ND | ND | ND | ND | ND | ND | ND | ND | ND | ND |
| 5318/4 | Human | 11/07/2016 | Blood | *Salmonella* Enteritidis | 24 | 30 | 30 | 30 | 23 | 30 | 26 | 25 | 27 | 36 | ND | ND | 2 | 0,012 |
| 5353/4 | Human | 29/07/2016 | Blood | *Salmonella* Enteritidis | 10 | 10 | 33 | 29 | 10 | 30 | 23 | 24 | 10 | 35 | ND | ND | 2 | 0,012 |
| 5376/4 | Human | 10/08/2016 | Blood | *Salmonella* Typhimurium | 10 | s | 33 | 32 | 10 | 27 | 24 | 10 | 10 | 36 | ND | ND | 2 | 0,012 |
| 5390/4 | Human | 16/08/2016 | Blood | *Salmonella* Typhimurium | 10 | 10 | 17 | 22 | 10 | 18 | 20 | 10 | 10 | 35 | ND | ND | 96 | 0,5 |
| 5403/4 | Human | 27/08/2016 | Blood | *Salmonella* Typhimurium | 10 | 10 | 33 | 29 | 10 | 29 | 25 | 10 | 10 | 34 | ND | ND | 2 | 0,012 |
| 5464/4 | Human | 23/09/2016 | Blood | *Salmonella* Typhimurium | 10 | 10 | 32 | 30 | 17 | 30 | 25 | 10 | 10 | 36 | ND | ND | 2 | 0,012 |
| 5548/4 | Human | 28/10/2016 | Blood | *Salmonella* Typhimurium | 10 | 10 | 31 | 28 | 10 | 30 | 25 | 23 | 25 | 35 | ND | ND | 1.5 | 0,008 |
| 5561/4 | Human | 05/11/2016 | Blood | *Salmonella* Typhi | ND | ND | ND | ND | ND | ND | ND | ND | ND | ND | ND | ND | ND | ND |
| 5566/4 | Human | 07/11/2016 | Blood | *Salmonella* Enteritidis | 10 | 10 | 29 | 27 | 10 | 27 | 23 | 24 | 10 | 33 | ND | ND | 3 | 0,012 |
| 5568/4 | Human | 08/11/2016 | Blood | *Salmonella* Enteritidis | 10 | 10 | 33 | 29 | 10 | 31 | 24 | 25 | 10 | 35 | ND | ND | 1.5 | 0,008 |
| 5578/4 | Human | 15/11/2016 | Blood | *Salmonella* Enteritidis | 10 | 10 | 31 | 29 | 10 | 31 | 24 | 25 | 25 | 34 | ND | ND | 2 | 0,006 |
| 5590/4 | Human | 18/11/2016 | Blood | *Salmonella* Enteritidis | 10 | 10 | 31 | 28 | 10 | 29 | 24 | 25 | 10 | 34 | ND | ND | 2 | 0,008 |
| 5598/4 | Human | 22/11/2016 | Blood | *Salmonella* Typhimurium | 10 | 10 | 34 | 29 | 10 | 29 | 25 | 10 | 10 | 33 | ND | ND | 2 | 0,008 |
| 5601/4 | Human | 24/11/2016 | Blood | *Salmonella* Typhimurium | 10 | 10 | 34 | 30 | 10 | 30 | 26 | 10 | 10 | 35 | ND | ND | 2 | 0,008 |
| 5699/4 | Human | 31/01/2017 | Blood | *Salmonella* Enteritidis | 10 | 10 | 32 | 27 | 10 | 26 | 24 | 27 | 10 | 34 | 10 | 10 | 2 | 0,016 |
| 5809/4 | Human | 13/05/2017 | Blood | *Salmonella* Typhimurium | 10 | 10 | 33 | 28 | 10 | 29 | 25 | 10 | 10 | 36 | ND | ND | 2 | 0,012 |
| 5810/4 | Human | 13/05/2017 | Blood | *Salmonella* Typhimurium | 10 | 10 | 34 | 29 | 10 | 28 | 26 | 10 | 10 | 34 | ND | ND | 2 | 0,008 |
| 5835/4 | Human | 31/05/2017 | Blood | *Salmonella* Typhimurium | 10 | 10 | 30 | 27 | 10 | 28 | 24 | 10 | 10 | 35 | 10 | 10 | 1 | 0,012 |
| 5899/4 | Human | 29/08/2017 | Blood | *Salmonella* Enteritidis | 10 | 10 | 32 | 29 | 10 | 28 | 24 | 24 | 10 | 35 | 10 | 10 | 1.5 | 0,016 |
| 6001/4 | Human | 06/06/2018 | Blood | *Salmonella* Paratyphi C | 10 | 10 | 34 | 29 | 10 | 29 | 23 | 10 | 10 | 38 | 10 | 10 | 1 | 0,016 |
| 6014/4 | Human | 09/06/2018 | Blood | *Salmonella* Enteritidis | 10 | 10 | 32 | 28 | 10 | 29 | 24 | 23 | 10 | 34 | 10 | 10 | 1,5 | 0,012 |
| 6111/4 | Human | 02/07/2018 | Blood | *Salmonella* Typhi | ND | ND | ND | ND | ND | ND | ND | ND | ND | ND | ND | ND | ND | ND |
| 6155/4 | Human | 08/07/2018 | Blood | *Salmonella* I.6,7y:- | 26 | 30 | 34 | 29 | 24 | 27 | 24 | 24 | 26 | 34 | 30 | 28 | 1 | 0,008 |
| 6195/4 | Human | 16/07/2018 | Blood | *Salmonella* Typhi | ND | ND | ND | ND | ND | ND | ND | ND | ND | ND | ND | ND | ND | ND |
| 6213/4 | Human | 17/07/2018 | Blood | *Salmonella* Enteritidis | 10 | 10 | 34 | 29 | 10 | 30 | 23 | 24 | 10 | 34 | 10 | 10 | 1,5 | 0,012 |
| 6281/4 | Human | 03/08/2018 | Blood | *Salmonella* species | ND | ND | ND | ND | ND | ND | ND | ND | ND | ND | ND | ND | ND | ND |
| 6284/4 | Human | 07/08/2018 | Blood | *Salmonella* Typhimurium | 10 | 10 | 32 | 29 | 10 | 29 | 25 | 10 | 10 | 36 | 10 | 10 | 1,5 | 0,012 |
| 6332/4 | Human | 11/08/2018 | Blood | *Salmonella* Enteritidis | 10 | 10 | 38 | 28 | 10 | 30 | 24 | 25 | 10 | 38 | 10 | 10 | 1 | 0,008 |
| 6436/4 | Human | 06/09/2018 | Blood | *Salmonella* Typhimurium | 10 | 10 | 29 | 26 | 10 | 28 | 22 | 10 | 10 | 34 | 10 | 10 | 1 | 0,023 |
| 6803/4 | Human | 20/11/2018 | Blood | *Salmonella* Typhi | 10 | 10 | 32 | 29 | 10 | 19 | 10 | 24 | 26 | 36 | 10 | 10 | 1 | 0,25 |
| 6862/4 | Human | 09/12/2018 | Blood | *Salmonella* Typhi | ND | ND | ND | ND | ND | ND | ND | ND | ND | ND | ND | ND | ND | ND |
| 6892/4 | Human | 27/12/2018 | Blood | *Salmonella* Typhi | 10 | 10 | 30 | 30 | 10 | 20 | 10 | 22 | 28 | 36 | 10 | 10 | 1 | 0,19 |
| 7279/4 | Human | 10/06/2019 | Blood | *Salmonella* Typhimurium | 10 | 10 | 17 | 21 | 24 | 19 | 20 | 10 | 10 | 33 | 10 | 10 | 256 | 0,19 |
| 7281/4 | Human | 13/06/2019 | Blood | *Salmonella* Typhimurium | 10 | 10 | 19 | 22 | 24 | 16 | 16 | 11 | 10 | 34 | 10 | 10 | 128 | 0,125 |
| 7292/4 | Human | 14/06/2019 | Blood | *Salmonella* Typhimurium | 10 | 10 | 20 | 20 | 23 | 27 | 21 | 23 | 10 | 33 | 10 | 10 | 192 | 0,012 |
| AB005FOA1 | Rat | 05/09/2016 | Liver | *Salmonella* Enteritidis | 23 | 30 | 30 | 28 | 24 | 30 | 22 | 26 | 25 | 34 | ND | ND | 2 | 0,012 |
| AB005FOA3 | Rat | 06/09/2016 | Liver | *Salmonella* Dublin | 24 | 30 | 32 | 27 | 22 | 27 | 23 | 26 | 25 | 36 | ND | ND | 1.5 | 0,023 |
| AB006FOA3 | Rat | 05/09/2016 | Liver | *Salmonella* Enteritidis | 22 | 31 | 32 | 27 | 24 | 25 | 23 | 26 | 24 | 36 | ND | ND | 3 | 0,023 |
| MC004SEA1 | Rat | 18/04/2016 | Rectal content | *Salmonella* II:42:r:- | 25 | 31 | 31 | 30 | 24 | 29 | 28 | 24 | 25 | 38 | ND | ND | 2 | 0,012 |
| MC019SEA2 | Rat | 08/05/2016 | Rectal content | *Salmonella* II:42:r:- | 25 | 32 | 31 | 30 | 23 | 29 | 28 | 24 | 24 | 34 | ND | ND | 2 | 0,012 |
| MC020SEA1 | Rat | 08/05/2016 | Rectal content | *Salmonella* II:42:r:- | 25 | 32 | 34 | 30 | 25 | 33 | 26 | 24 | 24 | 34 | ND | ND | 3 | 0,008 |
| MC025FOA1 | Rat | 15/05/2016 | Liver | *Salmonella* Typhimurium | 28 | 31 | 30 | 30 | 28 | 28 | 26 | 25 | 25 | 36 | ND | ND | 2 | 0,012 |
| MC035SEA2 | Rat | 12/06/2016 | Rectal content | *Salmonella* II:42:r:- | 25 | 31 | 31 | 29 | 25 | 29 | 26 | 25 | 25 | 36 | ND | ND | 2 | 0,016 |
| MC048RAA1 | Rat | 20/09/2016 | Spleen | *Salmonella* Enteritidis | 24 | 32 | 30 | 28 | 22 | 28 | 21 | 26 | 25 | 33 | ND | ND | 2 | 0,012 |
| MC053RAA1 | Rat | 20/09/2016 | Spleen | *Salmonella* Enteritidis | 25 | 31 | 32 | 29 | 23 | 30 | 23 | 25 | 25 | 35 | ND | ND | 2 | 0,012 |
| MC054FOA1 | Rat | 20/09/2016 | Liver | *Salmonella* Enteritidis | 26 | 33 | 33 | 30 | 23 | 28 | 23 | 28 | 27 | 36 | ND | ND | 3 | 0,012 |
| MC071FOA1 | Rat | 06/10/2016 | Liver | *Salmonella* Typhimurium | 10 | 10 | 33 | 30 | 10 | 29 | 26 | 25 | 23 | 34 | 10 | 30 | 1 | 0,012 |
| MC071RAB5 | Rat | 06/10/2016 | Spleen | *Salmonella* Weltevreden | 25 | 31 | 34 | 28 | 27 | 29 | 24 | 23 | 25 | 34 | 32 | 30 | 1 | 0,012 |
| MC080SEA2 | Rat | 29/11/2016 | Rectal content | *Salmonella* Weltevreden | 24 | 32 | 34 | 30 | 28 | 28 | 22 | 25 | 23 | 34 | ND | ND | 1.5 | 0,008 |
| MC081SEA3 | Rat | 29/11/2016 | Rectal content | *Salmonella* Weltevreden | 25 | 32 | 30 | 30 | 28 | 30 | 25 | 25 | 25 | 36 | ND | ND | 1.5 | 0,008 |
| MC083RAB2 | Rat | 29/11/2016 | Spleen | *Salmonella* Weltevreden | 25 | 32 | 32 | 32 | 28 | 30 | 25 | 25 | 25 | 36 | ND | ND | 1.5 | 0,006 |
| MR002SEA1 | Rat | 11/05/2016 | Rectal content | *Salmonella* Orion | 26 | 31 | 32 | 32 | 27 | 29 | 25 | 24 | 26 | 36 | ND | ND | 1.5 | 0,008 |
| MR002SEA2 | Rat | 11/05/2016 | Rectal content | *Salmonella* II:42:r:- | 25 | 28 | 32 | 30 | 20 | 29 | 24 | 24 | 23 | 32 | ND | ND | 4 | 0,012 |
| MT029FOA1 | Rat | 28/11/2016 | Liver | *Salmonella* Weltevreden | 25 | 31 | 34 | 30 | 30 | 30 | 25 | 25 | 25 | 35 | ND | ND | 0.75 | 0,006 |
| MY025SEA3 | Rat | 18/05/2016 | Rectal content | *Salmonella* Kapemba | 25 | 27 | 30 | 25 | 23 | 26 | 23 | 22 | 22 | 33 | ND | ND | 3 | 0,016 |
| MY030FOA4 | Rat | 18/05/2016 | Liver | *Salmonella* Weltevreden | 26 | 30 | 30 | 28 | 23 | 28 | 26 | 24 | 24 | 30 | ND | ND | 2 | 0,016 |
| MY055SEB1 | Rat | 14/06/2016 | Rectal content | *Salmonella* II:42:r:- | 27 | 30 | 32 | 30 | 25 | 29 | 28 | 23 | 25 | 34 | ND | ND | 3 | 0,012 |
| MY063SEA5 | Rat | 24/07/2016 | Rectal content | *Salmonella* Weltevreden | 25 | 30 | 30 | 28 | 24 | 29 | 25 | 24 | 23 | 33 | ND | ND | 1 | 0,008 |
| MY100FOA1 | Rat | 26/09/2016 | Liver | *Salmonella* Enteritidis | 23 | 30 | 30 | 28 | 23 | 29 | 24 | 25 | 26 | 34 | ND | ND | 2 | 0,023 |
| MY101RAA1 | Rat | 26/09/2016 | Spleen | *Salmonella* Enteritidis | 24 | 32 | 28 | 28 | 25 | 29 | 26 | 27 | 25 | 36 | ND | ND | 2 | 0,016 |
| MY102RAA1 | Rat | 26/09/2016 | Spleen | *Salmonella* Enteritidis | 24 | 34 | 30 | 28 | 24 | 31 | 26 | 25 | 25 | 34 | ND | ND | 3 | 0,012 |
| MY109FOA1 | Rat | 26/09/2016 | Liver | *Salmonella* Weltevreden | 24 | 30 | 30 | 30 | 26 | 29 | 26 | 24 | 24 | 33 | ND | ND | 2 | 0,023 |
| MY123FOA1 | Rat | 31/10/2016 | Liver | *Salmonella* Weltevreden | 20 | 31 | 32 | 30 | 26 | 32 | 24 | 26 | 26 | 36 | ND | ND | 1 | 0,008 |
| MY143FOA1 | Rat | 22/11/2016 | Liver | *Salmonella* Weltevreden | 25 | 32 | 30 | 30 | 28 | 29 | 23 | 25 | 22 | 34 | ND | ND | 1 | 0,008 |
| MY186SEA1 | Rat | 27/03/2017 | Rectal content | *Salmonella* II:42:r:- | 25 | 31 | 33 | 30 | 24 | 31 | 25 | 25 | 24 | 38 | ND | ND | 2 | 0,008 |
| MY213RAA1 | Rat | 10/06/2018 | Spleen | *Salmonella* Typhimurium | 25 | 30 | 30 | 27 | 23 | 26 | 24 | 25 | 24 | 30 | 28 | 28 | 1,5 | 0,012 |
| MY213RAB1 | Rat | 10/06/2018 | Spleen | *Salmonella* II:42:r:- | 24 | 29 | 28 | 28 | 23 | 28 | 25 | 23 | 23 | 30 | 30 | 24 | 1 | 0,016 |
| MY218FOA1 | Rat | 10/06/2018 | Liver | *Salmonella* II:42:r:- | 24 | 30 | 31 | 29 | 24 | 28 | 25 | 24 | 22 | 33 | 28 | 25 | 0,75 | 0,012 |
| MY218FOA2 | Rat | 10/06/2018 | Liver | *Salmonella* Typhimurium | 23 | 29 | 30 | 26 | 24 | 27 | 21 | 23 | 22 | 33 | 25 | 26 | 1 | 0,012 |
| MY300FOA2 | Rat | 08/10/2018 | Liver | *Salmonella* Typhimurium | 24 | 30 | 33 | 28 | 25 | 28 | 24 | 24 | 24 | 35 | 30 | 27 | 1 | 0,012 |
| MY302RAA1 | Rat | 08/10/2018 | Spleen | *Salmonella* Typhimurium | 10 | 10 | 32 | 30 | 10 | 28 | 24 | 10 | 10 | 34 | 10 | 10 | 3 | 0,012 |
| MY305FOA1 | Rat | 08/10/2018 | Liver | *Salmonella* Typhimurium | 10 | 10 | 28 | 25 | 10 | 25 | 22 | 10 | 10 | 33 | 10 | 10 | 2 | 0,016 |
| MY305FOB4 | Rat | 08/10/2018 | Liver | *Salmonella* II:42:r:- | 23 | 29 | 31 | 27 | 22 | 27 | 25 | 23 | 22 | 32 | 22 | 25 | 2 | 0,012 |
| MY318SEA1 | Rat | 28/10/2018 | Rectal content | *Salmonella* II:42:r:- | 22 | 30 | 30 | 24 | 23 | 26 | 23 | 23 | 21 | 30 | 32 | 25 | 2 | 0,012 |
| MY330FOB4 | Rat | 04/11/2018 | Liver | *Salmonella* Kapemba | 24 | 28 | 30 | 26 | 24 | 24 | 22 | 24 | 22 | 32 | 22 | 26 | 1,5 | 0,012 |
| MY331FOA2 | Rat | 04/11/2018 | Liver | *Salmonella* II:42:r:- | 24 | 31 | 32 | 28 | 24 | 29 | 25 | 23 | 23 | 34 | 33 | 28 | 1,5 | 0,012 |
| MY331RAA1 | Rat | 04/11/2018 | Spleen | *Salmonella* Kapemba | 24 | 31 | 31 | 28 | 25 | 29 | 24 | 23 | 24 | 34 | 28 | 29 | 2 | 0,016 |
| MY332FOA1 | Rat | 04/11/2018 | Liver | *Salmonella* Kapemba | 24 | 30 | 29 | 26 | 21 | 25 | 21 | 22 | 23 | 31 | 22 | 26 | 1,5 | 0,012 |
| MY338FOA1 | Rat | 11/11/2018 | Liver | *Salmonella* Kapemba | 24 | 30 | 30 | 26 | 22 | 27 | 22 | 24 | 23 | 36 | 30 | 28 | 0,75 | 0,012 |
| MY347SAA1 | Rat | 18/11/2018 | Blood | *Salmonella* II:42:r:- | 24 | 30 | 31 | 29 | 25 | 26 | 24 | 24 | 22 | 32 | 32 | 25 | 2 | 0,012 |
| MY355FOA2 | Rat | 18/11/2018 | Liver | *Salmonella* II:42:r:- | 24 | 29 | 30 | 26 | 21 | 27 | 24 | 24 | 20 | 30 | 27 | 25 | 2 | 0,008 |
| MY355FOB5 | Rat | 18/11/2018 | Liver | *Salmonella* Kapemba | 24 | 30 | 31 | 27 | 25 | 27 | 23 | 22 | 23 | 33 | 30 | 28 | 1,5 | 0,016 |
| MY356FOA1 | Rat | 18/11/2018 | Liver | *Salmonella* II:42:r:- | 24 | 31 | 31 | 28 | 23 | 28 | 25 | 23 | 24 | 34 | 34 | 27 | 2 | 0,012 |
| MY356RAA1 | Rat | 18/11/2018 | Spleen | *Salmonella* Kapemba | 24 | 29 | 30 | 27 | 25 | 25 | 22 | 23 | 20 | 30 | 23 | 26 | 1,5 | 0,012 |
| MY359FOB4 | Rat | 16/12/2018 | Liver | *Salmonella* Kapemba | 24 | 30 | 31 | 27 | 26 | 29 | 24 | 24 | 25 | 33 | 29 | 28 | 1,5 | 0,016 |
| MY361RAA2 | Rat | 16/12/2018 | Spleen | *Salmonella* Kapemba | 23 | 27 | 30 | 25 | 23 | 22 | 22 | 22 | 21 | 31 | 24 | 26 | 1 | 0,012 |
| MY365RAA3 | Rat | 16/12/2018 | Spleen | *Salmonella* Kapemba | 23 | 28 | 29 | 26 | 25 | 28 | 23 | 24 | 22 | 33 | 26 | 27 | 1,5 | 0,016 |
| MY365RAB2 | Rat | 16/12/2018 | Spleen | *Salmonella* Mikawasima | 24 | 31 | 30 | 28 | 23 | 26 | 24 | 22 | 24 | 32 | 26 | 26 | 2 | 0,012 |
| MY365SEA1 | Rat | 16/12/2018 | Rectal content | *Salmonella* II:42:r:- | 23 | 29 | 30 | 26 | 23 | 25 | 24 | 23 | 23 | 33 | 32 | 27 | 2 | 0,012 |
| MY365SEB4 | Rat | 16/12/2018 | Rectal content | *Salmonella* Typhimurium | 24 | 29 | 30 | 28 | 24 | 24 | 22 | 20 | 21 | 32 | 24 | 26 | 1 | 0,012 |
| MY373FOA3 | Rat | 23/12/2018 | Liver | *Salmonella* Typhimurium | 22 | 28 | 30 | 25 | 24 | 25 | 20 | 23 | 20 | 30 | 22 | 24 | 1 | 0,012 |
| MY373FOA4 | Rat | 23/12/2018 | Liver | *Salmonella* II:42:r:- | 23 | 29 | 30 | 28 | 22 | 26 | 24 | 23 | 21 | 32 | 30 | 26 | 2 | 0,012 |
| MY375RAB1 | Rat | 23/12/2018 | Spleen | *Salmonella* II:42:r:- | 24 | 30 | 30 | 26 | 22 | 25 | 23 | 21 | 20 | 32 | 26 | 26 | 1,5 | 0,012 |
| MY375SAA1 | Rat | 23/12/2018 | Blood | *Salmonella* Typhimurium | 22 | 30 | 30 | 28 | 25 | 27 | 24 | 23 | 23 | 34 | 30 | 28 | 1,5 | 0,012 |
| MY375SEB4 | Rat | 23/12/2018 | Rectal content | *Salmonella* Kapemba | 22 | 27 | 30 | 24 | 24 | 28 | 22 | 23 | 21 | 32 | 26 | 27 | 2 | 0,016 |
| MY377SEA1 | Rat | 23/12/2018 | Rectal content | *Salmonella* Kapemba | 23 | 29 | 32 | 27 | 25 | 27 | 22 | 23 | 22 | 32 | 28 | 28 | 2 | 0,016 |
| MY377SEA4 | Rat | 23/12/2018 | Rectal content | *Salmonella* Mikawasima | 24 | 30 | 30 | 29 | 25 | 30 | 24 | 25 | 22 | 34 | 30 | 29 | 1,5 | 0,012 |
