# Supplementary material for: Urban rats as carriers of invasive Salmonella Typhimurium sequence type 313, Kisangani, Democratic Republic of Congo
Source: PLoS Negl Trop Dis. 2022 Sep 6;16(9):e0010740. doi: 10.1371/journal.pntd.0010740 (PMC9481155; doi:10.1371/journal.pntd.0010740)
Supplement: S1 Fig — (DOCX) [file pntd.0010740.s004.docx]

**Supplemental Figure 1:** Timeline of human and rat sampling. Human sampling (blood cultures) was one during the entire period, rat sampling (outreach visits to marketplaces) was interrupted during the grey-shaded periods. Numbers refer to humans with *Salmonella* bloodstream infection and to rats carrying *Salmonella*. Rat drawing by Francisca Arévalo, patient drawing by Adrian Cocquet from NounProject.com.

**
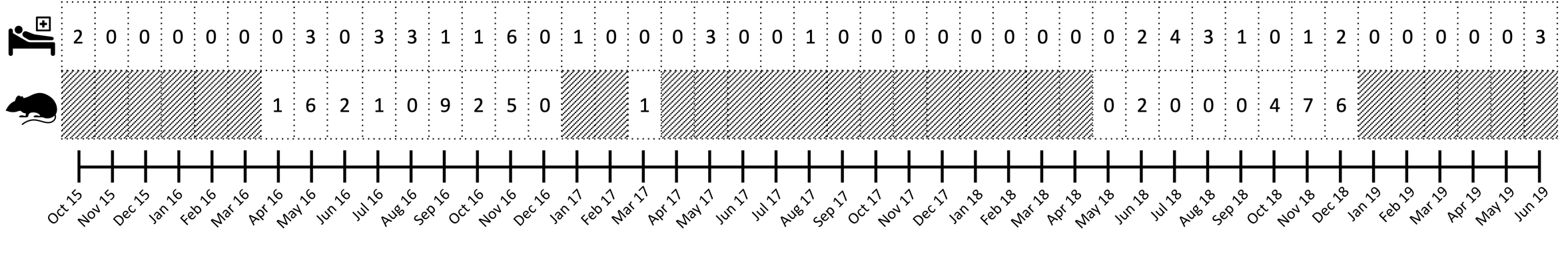
**
