# Supplementary material for: Urban rats as carriers of invasive Salmonella Typhimurium sequence type 313, Kisangani, Democratic Republic of Congo
Source: PLoS Negl Trop Dis. 2022 Sep 6;16(9):e0010740. doi: 10.1371/journal.pntd.0010740 (PMC9481155; doi:10.1371/journal.pntd.0010740)
Supplement: S2 Fig — (DOCX) [file pntd.0010740.s005.docx]

**Supplemental Figure 2.**  **Clustering of human *Salmonella* Enteritidis isolates with ST11 *Salmonella* Enteritidis isolates described by Feasey et al. [1]** { **according to HC50 (cgMLST V2 and hierBAPS).**

Minimum spanning tree (MSTree V2) comparing the ST11 genomes analysed by Feasey et al. in 2016 and the genomes in this study. The genomes of *S.* Enteritidis ST11 in this study (circled in red) share HC50_12675 with those genomes available on EnteroBase from the study from Feasey et al., that belong to hierBAPS 9 cluster (B).

B.

A.

References

1. Feasey NA, Hadfield J, Keddy KH, Dallman TJ, Jacobs J, Deng X, et al. Distinct Salmonella Enteritidis lineages associated with enterocolitis in high-income settings and invasive disease in low-income settings. Nature Genetics. 2016;48: 1211–1217. doi:10.1038/ng.3644
