# Supplementary material for: Urban rats as carriers of invasive Salmonella Typhimurium sequence type 313, Kisangani, Democratic Republic of Congo
Source: PLoS Negl Trop Dis. 2022 Sep 6;16(9):e0010740. doi: 10.1371/journal.pntd.0010740 (PMC9481155; doi:10.1371/journal.pntd.0010740)
Supplement: Attachment — Submitted filename: 220718 Response to the reviewer.docx [file pntd.0010740.s006.docx]

Dear editor and reviewers,

Firstly, thank you very much for your thorough and constructive review. We have tried to answer all your questions and comments in the letter below and in the manuscript text. For your information, the line number referred to are the line numbers from the track changes-documents.

**General comments by the editor**

*Please note editorial comments made by reviewer 1, along with suggestions for more detail on the rodent trapping methods used and defining “trapping success” in the methods section of the revised manuscript.*

The method section was changed based on the comments. Details about the different changes are explained below for each specific comment.

*Both reviewers 1 & 3 asked for a map with rodent trapping sites indicated.*

We have now included a map with rodent trapping sites (figure 1, referred to in L182).

*Reviewer 3 also requested an excel file to include data used from other groups/public databases. A separate excel file with detailed MIC data should also be provided with the revised manuscript.*

We have included two supplementary tables to answer to these comments.

*All genomic data used in this study will need to be made available per PLoS NTDs guidelines.*

The genomic data is available on the ENA depository (<https://www.ebi.ac.uk/ena/browser/view/PRJEB54047>)

**Reviewer #1 comments:**

1. *Sometimes nontyphoidal Salmonella (NTS) is qualified with ‘invasive’ unnecessarily or inappropriately. For example, as a redundancy when bloodstream isolates are being described, and perhaps incorrectly when carriage isolates are being described (i.e., isolates from rectal content, when a definition for invasion is not met).*

Thank you for addressing this oversight. We have adapted this throughout the full manuscript.

*2. The term ‘pan-susceptible’ is used without definition. Relatedly, a full listing of antimicrobial agents tested is not provided in the Methods.*

We have added a definition of ‘pan-susceptible’ in the text (L648). In addition, we have now added a supplemental file with all AST results, including the full listing of the antimicrobial agents tested.

*3. There seems to be some conflation of carriage, infection, and disease terms in relation to rat isolates, with 'carriage' being used when infection or disease is not comprehensively* excluded.

We have carefully reviewed the text again to ensure the correct use of the terms carriage/disease/infection.

*4. Please check all occurrences of the word ‘rate.’ On several occasions the word ‘rate’ is used to describe a proportion or prevalence, rather than a time-denominated measure of incidence. For example, ‘case fatality rate’ is used when ‘case fatality ratio’ would be more appropriate, and ‘high rate of antimicrobial resistance’ is used when ‘high prevalence of antimicrobial resistance’ is intended. A lot has been written about this (pedantic) point, but one recent example is by S Mantha 'Ratio, rate, or risk?' Lancet Infect Dis 2020; 21: 165-6.*

Thank you for this remark and explanation. The text was adapted accordingly.

*5. Oxford (serial) commas would be helpful for clarity in places.*

Oxford commas have been added were appropriate.

*6. Nontyphoidal Salmonella cause in humans, anatomically and pathologically, predominantly entercolitis rather than gastroenteritis. If you wish to be anatomically specific, I would switch terms to enterocolitis. If you wish to avoid this issue, perhaps a clinical term like ‘diarrhea’ would be more accurate.*

Thank you for this remark and explanation. The text was adapted accordingly.

*7. ‘Paratyphi’ is not a recognized serovar of Salmonella enterica, but Salmonella Paratyphi A, Paratyphi B, and Paratyphi C are.*

We have now changed the text to “*Salmonella* Paratyphi A (L63).

*8. It would be useful to say a little more about non-genomic evidence for NTS adaptation to humans in the Introduction.*

We have added a sentence and references in L64-66 “These invasive NTS are clinically specific: they are mainly causing bloodstream infections (and not diarrhea) and are associated with high mortality.”

*9. A little more detail on rodent trapping methods would be helpful. For example, site selection process, numbers of traps set per night per site, dates of trapping (in relation to seasons), whether trapping was outdoor, or indoor, or both, etc.*

Rat trapping dates were already available in supplemental table 1. The sites were selected based on a previous study (referred to in L182), we have added more details on the rat traps on L196-197: “The traps were installed in warehouses, rice mills, or sheltered places in the sampling sites, to prevent them from being stolen or misused.”

*10. What was the median (range) time in hours (or target) between trapping and euthanasia (i.e., lairage time)? What was the median (range) time in hours (or target) between euthanasia and necropsy? What storage conditions were rodents kept in after euthanasia and before necropsy? These points are relevant to onset of recent bacteremia in rats in prolonged lairage, and for postmortem translocation of organism from the gut to deep tissues.*

Additional details on the trapping, euthanasia and time between euthanasia and necropsy were given in L202-226: “At reception, rats were taken out of the trap using a single-use cloth bag for each animal. Still in the bag (to prevent scratches or bites), they were euthanized with an intraperitoneal injection of a lethal dose of ketamine (Ketalin, Shalina laboratories PVT LTD, Mumbai India, 0.5 - 1ml of 250mg/5ml solution). The delay between collection from the trapping site and euthanasia was less than 3 hours and the animal was dissected immediately after the euthanasia (less than 10 minutes).” And L236-237: “Internal organs collected were transported to the lab for preliminary analysis within 4 hours.”

*11. Little description of how ‘signs of illness’ in rats was assessed, nor of gross findings at rat necropsy are provided. Were gross findings besides length and weight recorded? I assume no histopathology of liver and spleen to rule out signs of inflammation?*

We have added a sentence on the (absence of) signs of illness in L362-363 “All captured rats were alert and active, and none showed signs of illness and weakness.”. Histopathology of liver and spleen were no objectives of the study, but deep organs were selected to evaluate the potential invasive infections (L564-568 in discussion).

*12. What was the rationale for selecting up to five colonies, rather than a higher or lower number?*

Advice on sampling was given by Mrs. Sophie Bertrand, formerly working in the National Sciensano reference laboratory for *Salmonella.* With a total of five colonies, the chances of isolating multiple serotypes (which is probable in rats) is higher. This was already explained in the discussion (L686-688) and in the following references: Lapuz et al, 2008 and Singh Suresh et al., 1980.

*13. A definition of a ‘carrier’ would be helpful. The case for calling isolation from spleen and liver ‘carriage’ is made, but I am concerned that they could represent disease isolates since histopathologic studies were not done, and details of gross necropsy findings were scanty in the report.*

The definition for this study was given in L129: “carriers (rats with *S.* Typhimurium or Enteritidis isolates from liver, spleen, blood, and/or intestinal content)”.

*14. The term ‘antibiotic’ is used when ‘antimicrobial’ may be more appropriate.*

We agree with the reviewer that ‘antimicrobial (resistance)’ is the preferred term and we consequently used this term except when giving technical details (antibiotic susceptibility testing methods and disk contents used).

*15. I suggest that numerators, proportions, and denominators be presented consistently in the conventional manner i.e., ‘x (y%) of z,’ or ‘Of z, x (y%)…’ Always give actual numbers for numerators and denominators. For example, I would avoid ‘one third’ as a proxy numerator.*

We have adapted this in the text where appropriate.

*16. ‘Trapping success’ is not defined in the Methods. Is this per trap, per site, or something else?*

Trapping success in now defined on L201 “Trapping success was defined as the proportion of rats caught per number of traps installed in the trapping site.”

*17. A map of greater Kisangani with trapping sites would be helpful. Adding relevant human participants would also be useful if geolocated and ethically acceptable.*

We have now included a map with rodent trapping sites (figure 1, referred to in L182).

*18. Since Salmonella Weltevreden was a common serovar isolated from rats, it would be useful to know whether the markets to which rodents had access in Kisangani sold fish, including farmed fish.*

The Yaté market has a section reserved for the sale of farmed fish, which is about 100 meters from the place where the traps were placed. Also in the central market, farmed fish is sold, but not in a specific section. These two markets sheltered most rats carrying *S.* Weltevreden. However, *S.* Weltevreden has not been described in fish in Africa up till now, so more research is needed for this, and this is out of the scope of this manuscript.

*19. More discussion on the interpretation of isolation of Salmonella from liver and spleen would be helpful. In particular, could this represent recent onset bacteremia following prolonged lairage, or post-mortem translocation from the gut to the deep tissues?*

This was already discussed in the discussed L563-570.

*20. The term ‘rat-to-rat’ transmission but be interpreted by some readers with an epidemiologic background as an indication of transmission by direct contact (c.f., fecally contaminated water or food, feces consumption, etc). I assume that is not the authors' intent, and wonder if ‘transmission among rats’ may be less likely to imply insights into the mode of transmission*.

We agree with the reviewer and have changed the terminology.

*21. It is likely going beyond the scope of the findings, but would the authors care to speculate on possible modes of transmission from rodents to people, should rodents be a reservoir here? For example, we are not told whether human slaughter and consumption of rats and other rodents is practiced in Kisangani. I assume that contamination of water and food by rodent feces is likely, but not explicitly mentioned.*

Potential transmission routes of rats to humans are listed in the discussion L572-615. For Kisangani in particular, we are aware that some species of rodents (specially the giant rat or cricetomys) are part of the usual menu of the population. There is no evidence of the consumption of Rattus, however, it has been reported during our field visits that thugs/orphans living in the various markets consume the rats there. But as this information is only circumstantial, we did not include it in the manuscript, since more research to confirm this possible route of transmission is needed.

**Reviewer #3 comments:**

*Line 107-108, how the sample calculation was conducted?*

Sample size calculation is now explained in more detail in L162-166: “Based on CLSI guidelines, a minimum sample size of 30 *Salmonella* spp. isolates was set for reliable data about serotype distribution and antimicrobial susceptibility profiles [28]. At an assumed NTS carrier ratio of 5 – 10% [29–32] we targeted to capture a total of 600 rats to have sample size of 30 rats carrying *Salmonella*.”

*Detailed excel sheet regarding the data used (from other groups or public datasets) are needed.*

This is now added as “supplemental table 1”.

*Is there any rat samples having multiple Salmonella isolates or serovars?*

Yes, this was already mentioned in table 2 (L387 – 392). A total of 73 single non-duplicate *Salmonella* serotype isolates were recovered from 46 rats; multiple serotypes were observed in 13 rats.

*Detailed excel sheet regarding the MIC data should also be included.*

This is now added as “supplemental table 3”

*Further serovar based analysis regarding Salmonella Dublin, II:42:r:-, Kapemba, Weltevreden is needed, some are important and potential hazards in Africa countries and many others.*

We agree with this comment, but since we have limited this research to iNTS (*S.* Typhimurium and *S.* Enteritidis) with major relevance for human disease. The other serovars are out of the scope of this manuscript.

*A map of sampling is needed.*

We have now included a map with rodent trapping sites (figure 1, referred to in L182).

*All genomic data is not available.*

The genomic data is available on the ENA depository (<https://www.ebi.ac.uk/ena/browser/view/PRJEB54047>)

*Rats as reservoirs of non-Typhoidal Salmonella, and many more references should be included.*

We agree that literature on rat as reservoirs of non-Typhoidal *Salmonella* is sparse. A thorough literature review was done before starting the writing process of this manuscript and all available references have been included.
